# Conceptual framework to advancing healthcare system through Agile methodologies, human factors engineering and adaptive leadership

**DOI:** 10.1108/JHOM-06-2025-0316

**Published:** 2026-01-27

**Authors:** Deepali Vishal Thombare, Jyothsna Mailapalli, Esther Omotola Adeyemi, Sharon Bommer

**Affiliations:** University of Dayton, Dayton, Ohio, USA

**Keywords:** Leadership, Agile methodologies, Healthcare system performance, Human factor engineering, Patient outcome

## Abstract

**Purpose:**

This paper explores how Agile methods, combined with human factors engineering and adaptive leadership, can improve healthcare delivery by enhancing system flexibility, workforce resilience and patient-centered care. As healthcare environments become more complex and rapidly changing, traditional rigid systems often fail to meet evolving needs. The paper identifies significant barriers to Agile adoption, including rigid organizational structures, limited resources and cultural resistance. It introduces an integrated solution, a Biometric Stress Monitoring Framework to reduce burnout and improve performance. This strategy supports the development of a flexible, responsive and sustainable healthcare system focused on quality outcomes.

**Design/methodology/approach:**

This paper applies a conceptual and theoretical analysis of leadership models, systems engineering and human factors. The framework presents a solution that align with clinical safety standards and organizational needs, offering practical guidance for implementing Agile principles in dynamic healthcare settings.

**Findings:**

Agile adoption strengthens healthcare system responsiveness, collaboration and adaptability. Key challenges include weak leadership capacity, fragmented infrastructure and resistance to cultural change. The proposed solutions support proactive stress management and leadership growth, improving staff well-being, better decision-making and higher quality in patient care delivery.

**Originality/value:**

This paper offers a novel perspective by connecting Agile principles with human-centered design and leadership development. The proposed approaches provide practical tools to support employee well-being and real-time responsiveness in healthcare. They contribute to addressing long-standing organizational challenges while advancing a more resilient and effective model of care.

## Introduction

Agile methodology is an iterative and flexible approach to leadership and problem-solving. Leaders do not attempt to impose fixed, technical solutions but instead create conditions for continuous adjustment, feedback and shared responsibility among teams. It contrasts sharply with traditional project management methodologies like the waterfall model, which often relies on fixed project plans and inflexible timelines. The agile methodology allows for ongoing collaboration among diverse stakeholders, including clinicians, patients and healthcare IT professionals, facilitating adaptive responses to complex healthcare demands (Neinstein *et al.*, 2016; Alotaibi and Almudhi, 2023). Yan (2014) explores human factors engineering, also known as ergonomics, as an evolved discipline that integrates human capabilities and limitations into designing and operating systems and products to optimize human well-being and overall system performance. The foundation of human factors engineering is rooted in a deep understanding of human physiological and psychological characteristics and their interplay with the work environment. It influences key design aspects such as workstation layouts, equipment usability and workflow optimization to enhance operational efficiency and employee wellness. Human factors engineering enhances Agile adoption in healthcare by ensuring that adaptation to changing clinical demands is aligned with usability, error reduction and cognitive workload considerations (Badiru and Bommer, 2017; Improta *et al*., 2020). While Agile principles offer iterative improvement and stakeholder responsiveness suited to dynamic healthcare environments (Kokol *et al*., 2022; Holden *et al*., 2021), their translation into practice is hindered by hierarchical structures, compliance-heavy documentation and emerging technological complexity, such as AI-driven systems integration (Klemme *et al*., 2021; Mwaiseje and Mwagike, 2019). Aligning Agile responsiveness with human factors and regulatory constraints requires more than cultural advocacy; it demands a mechanism that carries out adaptability in real time.

Our research highlights critical barriers to healthcare system adaptability. Resilience-building programs for healthcare professionals are underfunded and poorly developed, leading to burnout and reduced productivity. Leadership development, particularly for middle and frontline managers, is insufficient, hindering learning, fostering resistance to change and disrupting teamwork (Forsgren *et al.*, 2022). Although Agile methodologies show potential, they are poorly aligned with healthcare needs, as legal requirements for extensive compliance documentation clash with Agile’s iterative approach, delaying improvements in patient care.

This paper aims, by means of a conceptual analysis of theoretical approaches presented and discussed in this paper, to develop integrated frameworks that combine Agile methodologies, human factors engineering and adaptive leadership to enhance the healthcare system and improve patient centered care in dynamic and high-stakes environments. The core contribution of this manuscript is the Biometric Stress Monitoring Framework, which integrates Agile management loops with stress biometrics to enable real-time workforce resilience decision.

## Background on leadership in Agile healthcare

In today’s rapidly evolving work environments, integrating agile management principles with transformational leadership, grounded in human factors engineering, provides a strategy for cultivating resilient, high-performing teams. Transformational leadership, defined by intellectual stimulation, idealized influence and individualized consideration, enables leaders to challenge established routines, articulate a clear value-driven vision and support the professional growth of team members (Gabel, 2013). Empirical studies confirm that this leadership style reduces burnout and promotes psychological empowerment in complex clinical and engineering settings (Chatterjee *et al*., 2018). These qualities align with Agile values, prioritizing iterative decision-making, rapid adjustment to change and team autonomy. Agile-oriented leaders facilitate adaptability by fostering open communication, continuous feedback loops and a learning culture where team members actively participate in process refinement. When paired with human factors engineering, this leadership approach becomes more operationally grounded, as it ensures that workflow adaptations account for cognitive load, ergonomics and system usability. Rather than treating Agile as a cultural concept alone, integrating these elements positions leadership as an active moderator between system-level agility and workforce well-being. Through this alignment, healthcare organizations can transition from static protocol-driven management toward a responsive, people-centered model capable of sustaining innovation and clinical quality.

### Improving healthcare system performance through leadership and Agile

Building leadership strategies enhances team effectiveness by directly addressing the psychological and social dynamics that shape workforce performance. Servant leadership has gained traction in healthcare and engineering environments due to its alignment with human-centered care principles and its emphasis on ethical responsibility, empathy and support in leader-follower relationships (Trastek *et al*., 2014). Unlike directive leadership models, servant leadership improves provider–patient interactions by reinforcing trust and emotional safety, which are critical in high-stakes care environments. This relational emphasis supports process optimization by attending to clinical outputs and emotional and cognitive demands placed on staff. Human factors in engineering complement this leadership style by examining workload balance, cognitive strain and communication flow, ensuring decision-making environments reduce friction and psychological fatigue. Elements such as autonomy, competence and relatedness, central to motivation theories, are actively supported under servant leadership models, resulting in higher engagement and sustained performance (Aij and Rapsaniotis, 2017). When these leadership behaviors intersect with Agile principles such as short feedback loops and adaptive team coordination, they create a climate that encourages safe experimentation, learning and collective problem-solving without fear of punitive consequences. This convergence of servant leadership, human factors engineering and Agile decision cycles establishes efficient workflows and a psychologically secure operational environment that absorbs stress without immediate performance degradation. This psychological safety and adaptive team structure form the foundation upon which resilience, as explored in the next section and leader support can be systematically developed rather than informally expected from individuals.

### Building resilience in Agile healthcare systems

Resilience is leveraging internal/external resources, safe environments and traits like self-efficacy during adversity (Giordano *et al*., 2022). According to this viewpoint, developing resilience is possible through focused interventions, as it is a dynamic process. Resilience-building programs have been shown to lower stress, anxiety and depression while raising staff self-efficacy, which makes them essential for healthcare personnel (Pipe *et al*., 2012). Employees’ general mental health is significantly increased by leaders who establish clear communication and foster a happy work environment (Baskin and Bartlett, 2021). Healthcare companies may strengthen their resilience by using agile approaches, enabling teams to adapt to emerging challenges and boost productivity. Although the value of resilience in healthcare is becoming more widely acknowledged, formal psychological therapies are still lacking in many institutions. Such tactics, for instance, have not been completely adopted by Italian healthcare systems, underscoring the continued need for methods to increase healthcare professionals, particularly in times of crisis (Wiig *et al*., 2020). Understanding and building resilience in healthcare professionals is crucial for their well-being and for guaranteeing safe and effective patient care as the healthcare landscape changes, especially in the aftermath of the COVID-19 outbreak. Over the course of five years and five different countries (Norway, England, the Netherlands, Australia, Japan and Switzerland), the Resilience in Healthcare (RiH) research program aims to deepen our understanding of healthcare quality through a comprehensive theoretical and practical framework of resilience (Wiig *et al*., 2020). This initiative addresses the need for models that help stakeholders and healthcare systems adjust to variations and disruptions. Investigating integrative frameworks, stakeholder participation and the dynamics of resilience across many healthcare contexts, the RiH program sets the groundwork for designing successful interventions that increase resilience at multiple levels. Research suggests that healthcare professionals encounter several obstacles, such as extended work schedules, insufficient safety gear and the psychological strain of patients with life-threatening illnesses.

## Agile vs traditional methodologies in healthcare

Healthcare is a complicated and uncertain industry and agile and traditional program management methodologies have advantages and disadvantages. Linear, plan-driven project management techniques have been the standard for healthcare businesses. Drawn from predictable sectors, these techniques emphasize planning, defined roles and strict adherence to budgets and timelines. These techniques work well for projects with defined goals and steady requirements. However, they are challenging to use in the healthcare industry because of the unpredictable nature of medical settings, the fast-changing demands of patients and shifting legal and regulatory contexts. As shown by the high failure rates of healthcare IT implementations, traditional approaches are often slow, rigid and ill-prepared to deal with unexpected challenges such as crises or technological advancements. As a result, project timelines are frequently extended, budgets are overrun and stakeholders are left unhappy (Kitzmiller *et al*., 2006).

On the other hand, Agile approaches, which were first created for the software sector, provide an adaptable substitute that is more in line with the changing healthcare landscape. Agile promotes more frequent value delivery and project turnaround times by emphasizing iterative development and continuous feedback, which may enhance stakeholder satisfaction and patient outcomes (Morsi *et al*., 2024). Agile methods are also more patient-centered since they involve all relevant parties, including patients and healthcare professionals, at every stage of the process, guaranteeing that the solutions created closely match their requirements and expectations (Kitzmiller *et al*., 2006). Agile’s less regulated methodology may be challenging to adopt in healthcare systems because of its deeply embedded hierarchies, intricate stakeholder networks and strict regulatory constraints (Morsi *et al*., 2024). Concerns are also raised about the viability of implementing. Agile’s supporters contend that, despite these drawbacks, the approach may still improve healthcare outcomes because of its benefits in adaptability, stakeholder interaction and quick feedback cycles, especially when customized to the particulars of the industry (Kitzmiller *et al*., 2006; Morsi *et al*., 2024).

## Challenges in Agile healthcare implementation

Despite the growing recognition of Agile’s value in healthcare transformation, its practical adoption remains fraught with systemic, cultural, technological and leadership-related challenges. The complexity of healthcare systems, rigid hierarchies, fragmented communication, limited funding and underdeveloped digital infrastructure create resistance to Agile principles. This section outlines critical barriers hindering Agile integration, including organizational culture, leadership deficiencies, broader structural and workforce management research limitations.

### Cultural and organizational resistance to Agile

Healthcare institutions face persistent cultural barriers when implementing Agile Innovation, particularly in environments where rigid hierarchies and compliance-centric mindsets dominate daily practice. While these models improve standardization, they are frequently criticized for prioritizing procedural efficiency over flexibility, limiting their usefulness in highly variable clinical settings (Holden *et al*., 2021). For Agile principles to take root, organizations must cultivate a psychologically safe environment where experimentation is permitted and minor process failures are treated as learning opportunities rather than compliance violations. Nevertheless, in many clinical teams, hesitation persists due to fear of deviation from approved protocols, limited autonomy and uncertainty regarding acceptable decision boundaries. This cultural hesitation is intensified when digital tools such as Agile Dashboards or Agile Treatment Plans introduce additional documentation layers, contributing to cognitive overload among practitioners. Disagreements over Key Performance Indicators (KPI) interpretation and a lack of standard operational language further fragment alignment, making it difficult for teams to converge around shared improvement objectives (Lakhani *et al*., 2020). While these cultural frictions manifest visibly in day-to-day practice, such as reluctance to experiment, documentation fatigue and reactive communication, they are not merely behavioral issues. They reflect a deeper misalignment between cultural expectations and the structural systems that govern decision-making authority, resource distribution and leadership responsiveness.

### Structural deficiencies and management barriers

The cultural resistance described above largely reflects broader structural deficiencies embedded in healthcare governance and resource management systems. Even when staff express willingness to adapt, adaptive behaviors cannot materialize without systemic mechanisms that enable real-time decision flexibility, such as authority to reassign workload, responsive resourcing, or digital infrastructure that supports live operational monitoring (Roblek *et al*., 2024). Many healthcare systems operate under centralized, heavy approval administrative structures shaped by political appointments, limited funding and bureaucratic accountability protocols. These conditions constrain clinical teams from insufficient staffing, outdated equipment and limited capacity to dynamically reallocate resources, even when local data signals emerging stress or patient surge conditions. Agile language is adopted rhetorically in such settings, but Agile capability remains structurally unsupported.

Leadership fragmentation further complicates adoption. While frontline teams are encouraged to be adaptive, senior management often controls workflow adjustments, creating a disconnect between strategy and operational execution. Without leadership structures that support delegated autonomy and rapid micro-adjustments, attempts to instill Agile culture remain superficial, which extends to incident response, where linear, protocol-based reaction models hinder adaptive learning during crises such as cyberattacks, staffing surges, or sudden resource depletion (He *et al*., 2022; Tolf *et al*., 2015). Low-resource and politically constrained healthcare environments face even steeper barriers, as governance frameworks do not allow middle-tier leaders to operationalize resilience through proactive stress mitigation or workload redistribution (Forsgren *et al*., 2022). These structural constraints demonstrate that Agile resistance in healthcare is not simply a cultural challenge but a management systems failure, where the absence of real-time decision authority and resource-responsive infrastructure prevents cultural intent from becoming operational practice.

### Workforce and human resources limitations in Agile adoption

Implementing Agile principles in healthcare is often restricted by rigid human resource systems prioritizing procedural compliance over adaptive receptiveness. Traditional human resources (HR) policies emphasize fixed role definitions, formal approval workflows and hierarchical decision-making, which slow staffing adjustments and limit the organization’s ability to redistribute workload when stress levels rise among clinical teams (Badiru and Bommer, 2017). In high-acuity environments, this rigidity reduces the ability of healthcare teams to respond dynamically to patient surges, contributing to fatigue and delayed care delivery. Trust and communication breakdowns between staff, management and patients further complicated Agile adoption. When employees perceive leadership decisions as detached from frontline conditions, engagement decreases and psychological safety remains low, which is critical for Agile responsiveness. Healthcare workers report disengagement when their stress signals and workload pressures are not acknowledged, reinforcing resistance to new adaptive workflows (Wilson and Balasundaram, 2024). This highlights the need for leadership systems that do not rely solely on scheduled reporting cycles but incorporate real-time workforce feedback loops, which the proposed biometric stress framework aims to facilitate.

Successfully embedding Agile practices into workforce management requires the dismantling of departmental silos and the creation of coordination mechanisms that link data, staff well-being indicators and task allocation decisions. Agile in this context is not simply a cultural value but a resource allocation capability, where staffing decisions are adjusted moment to match real clinical demand. Without this adaptive HR function, Agile remains rhetorical rather than operational, producing frustration among employees who are advised to “act flexibly” within structurally inflexible systems (Chakraborty *et al*., 2022). A further issue is the persistence of hierarchical career progression models in healthcare, where authority and adaptability are reserved for a few senior decision-makers. This limits the ability of middle-level leaders and supervisors to close the frontline stress to implement micro-adjustments in shift design, duty rotation, or cognitive load redistribution. Adaptive leadership capacity must be embedded at these intermediate levels rather than concentrated at the executive tier. However, current workforce structures, especially in training-intensive environments, still reward conformity and procedural accuracy over adaptive decision-making, limiting the emergence of Agile-ready leadership talent (Gabel, 2013; Demeke *et al*., 2024).

Research gaps also hinder Agile workforce transformation. Existing leadership studies in healthcare are heavily focused on transformational, or servant leadership applied at the cultural level, while micro-level adaptive decision practices, such as reallocating staff based on stress biomarker thresholds or using live dashboards to distribute cases, remain underexplored (Hanse *et al*., 2016). Funding limitations further restrict innovation in resilience modeling, resulting in a disconnect between theory and front-line workforce management practices (Jeffcott *et al*., 2009; Giordano *et al*., 2022).

## Benefits of Agile in healthcare

As healthcare systems become increasingly complex and dynamic, the need for adaptable, collaborative and patient-centered frameworks has never been greater. Integrating Agile into healthcare environments facilitates real-time decision-making, fosters multidisciplinary collaboration and supports the rapid adaptation of services to meet evolving patient and institutional needs. Implementing Agile practices enables healthcare organizations to deliver coordinated and high-quality care, from improving supply chain logistics and crisis response to strengthening team communication and leadership effectiveness. The following sections explore the wide-ranging benefits of Agile frameworks, demonstrating their impact on system performance, patient outcomes and workforce resilience across diverse healthcare contexts.

### Agile-driven care coordination and responsiveness

Agile approaches have been shown to have multiple benefits in the healthcare sector, including improved teamwork, real-time tracking and patient-centered treatment. Software development led to agile approaches prioritizing collaboration, adaptability and incremental progress. In medicine, technologies like the Agile Dashboard and Agile Treatment Plan (ATP) help team members stay consistently informed about patient status and treatment objectives. This mutual understanding improves communication between medical professionals and guarantees that treatment plans align with patients’ intended results. According to Lakhani *et al*. (2020), Agile approaches significantly enhance patient safety and care quality by displacing diverse, customized treatment plans that may result in misunderstandings and mistakes. Healthcare teams may develop a common knowledge of their goals by establishing clear KPIs within Agile frameworks. Teams may take a proactive and organized approach to patient care by formalizing goal setting and measurement, guaranteeing that the treatment process is effective and customized to meet each patient’s requirements (Holden *et al*., 2021).

Additionally, Agile’s intrinsic flexibility makes it possible to include a variety of stakeholders in the care process, which improves cooperation and insights and, eventually, results in better patient management. Slovenian hospitals have significantly benefited from adopting Agile management methods, improving effectiveness and patient care (Roblek *et al*., 2024). For example, Agile techniques allow firms to adjust to changes in the healthcare industry quickly brought about by technological breakthroughs and increased demands for high-quality treatment. Incorporating lean and agile approaches into pharmaceutical supply chains guarantees the availability of necessary drugs and resources. This strategy lowers costs through cooperative buying and inventory management and decreases treatment delays (Mishra *et al*., 2019). For temperature-sensitive items like insulin, where precise demand forecasting and regular replenishment are essential to avoiding shortages and waste, such a system enables more efficient management of cold supply chains, improving the overall effectiveness of healthcare delivery.

### Advancing sustainability in patient satisfaction

Integrating Agile and green practices in healthcare extensively improves patient satisfaction and service delivery (Chakraborty *et al*., 2022). Healthcare firms may emphasize patient needs and expectations by using a customer-centric strategy for providing high-quality services. This change improves the patient’s experience overall and raises satisfaction levels while fostering trust and positive connections between patients and providers. Initiatives for continuous improvement foster flexibility and efficiency, allowing healthcare institutions to evaluate and improve their procedures routinely. Thanks to this alignment, patient care is guaranteed to be sensitive to changing demands and expectations. Healthcare workers are empowered via open communication and group decision-making, which promotes a culture of accountability and responsibility and raises patient satisfaction. Stressing self-organizing teams also encourages cooperation among medical staff, which enhances care delivery coordination. Building trust inside the company is also essential because an open culture allows people to voice their opinions, worries and criticism without fear of reprisal, which promotes ongoing learning and development. These practices result in a more patient-centered healthcare environment, which makes hospitals competitive players in the rapidly growing medical tourism market. In addition to improving patient outcomes, healthcare companies may create a sustainable model that benefits patients and providers by combining green and Agile practices (Wilson and Balasundaram, 2024).

Using Agile concepts in incident response procedures in healthcare settings greatly improves operational effectiveness and service delivery. Assuring the preservation of crucial domain knowledge and forensic evidence necessary for precise incident analysis and resolution, the cooperation of multidisciplinary teams, including IT, operations, legal, human resources and forensic specialists, allows organizations to react to incidents quickly and efficiently (He *et al*., 2022). Agile techniques’ emphasis on the early restoration of essential services reduces patient care interruptions. It frees healthcare practitioners to concentrate on business-critical tasks that directly influence patient outcomes. Agile approaches promote a culture of continuous learning that motivates teams to assess their performance and adjust by favoring simple retrospectives over laborious documentation procedures (Tolf *et al*., 2015). Flexible allocation of resources improves incident response efficiency and fortifies healthcare organizations’ capacity to handle individual patient demands. Healthcare providers may swiftly adjust to unexpected occurrences, personnel shortages, or sudden spikes in demand by utilizing Agile techniques that help to create a more robust healthcare system that can handle the challenges of the modern, changing world.

### Leadership, resilience and adaptive crisis response

Heifetz conceptualizes Adaptive Leadership through six core pillars: getting on the balcony to gain strategic distance, identifying the adaptive challenge, regulating distress to keep pressure within a productive range, maintaining disciplined attention to prevent diversion from core issues, giving the work back to the people to foster ownership and protecting voices of leadership from below to ensure emerging insights are not suppressed. This model reorients leadership away from authority-driven direction toward the facilitation of collective adaptation under complexity, positioning leaders as orchestrators of learning rather than providers of technical solutions (Heifetz and Laurie, 2001). Rachmad (2022) reinforces this by framing adaptive leadership as a diagnostic process that distinguishes technical from adaptive problems and mobilizes stakeholders through iterative experimentation and systemic adjustment. Extending this into applied healthcare practice, Kuluski *et al*. (2021) demonstrate how distributing decision-making across providers, patients and caregivers enables person-centred responses to multifactorial challenges such as multimorbidity and structural determinants of care.

This paradigm aligns with resilience theory, which defines workforce stability not by the absence of strain but by the ability to detect and respond to stress signals before escalating into failure or burnout. In crisis settings, leadership becomes a response-based coordination of adaptive capacity, characterized by regulated distress, real-time redistribution of authority and preservation of feedback loops essential for situational learning. Empirical studies affirm this shift: Giordano *et al*. (2022) show that early recognition of strain strengthens system resilience, while Forsgren *et al*. (2022) report that localized adjustments such as shifting staffing patterns or pausing non-critical workloads reduce both clinical error rates and psychological burden even under resource constraints. Integrating biometric monitoring to detect physiological stress signals further operationalizes this approach, advancing adaptive leadership from reactive crisis management toward proactive regulation of workforce capacity as a measurable leadership function.

## Proposed solutions for Agile integration

To address the growing demands on healthcare systems and mitigate workforce strain, we propose two integrated frameworks designed to enhance operational agility, staff well-being and leadership reactiveness. This paper follows a conceptual systems design methodology which is appropriate for early-stage model innovation in healthcare because it allows for the initial exploration and synthesis of interdisciplinary theories such as Agile adaptability, human factors ergonomics and adaptive leadership without the immediate need for empirical data, which may be impractical at this emerging stage due to resource constraints, ethical considerations and the complexity of healthcare environments. This approach facilitates the creation of a robust theoretical foundation that can guide subsequent empirical testing and practical implementation, ensuring the framework’s feasibility and relevance across the healthcare system before committing to costly trials. Rather than empirical testing, the design process abstracts theoretical constructs into decision nodes, feedback loops and trigger responses.

### Biometric Stress Monitoring Framework

**Plate 1:**
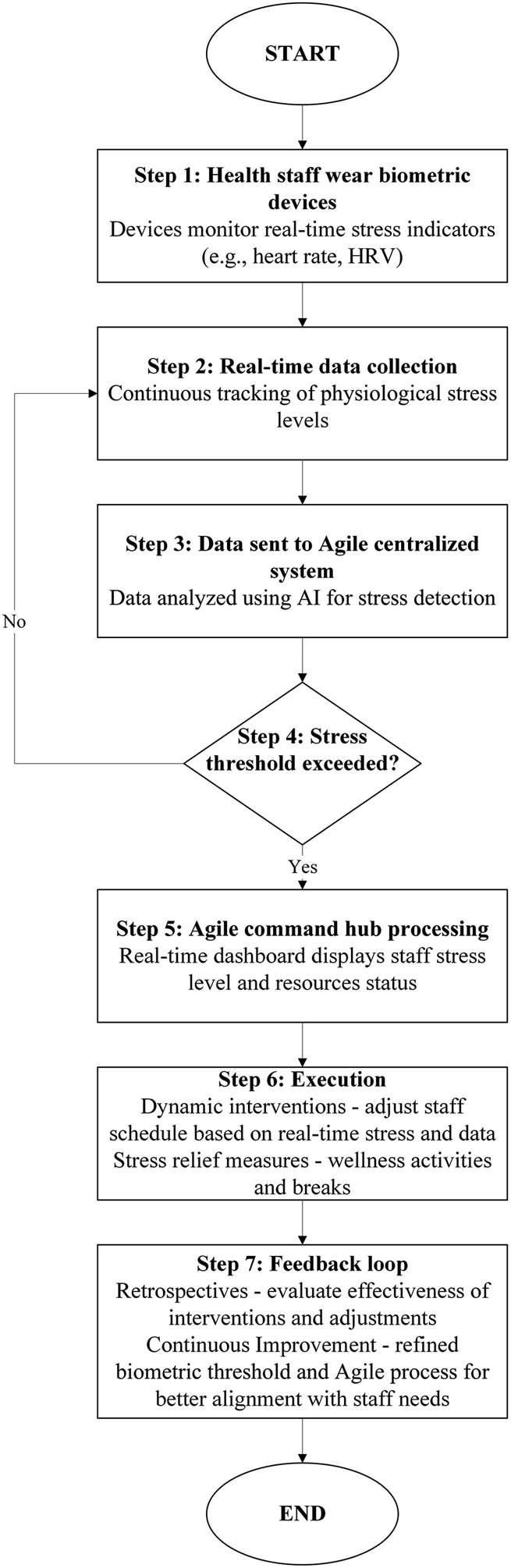
Biometric stress monitoring framework application in healthcare. Sources: Authors’ own work

Plate 1 illustrates the proposed Biometric Stress Monitoring Framework for managing healthcare staff stress functions as a complete, adaptable system, incorporating biometric stress monitoring, real-time data analytics and Agile approaches to promote staff well-being. A significant increase in heart rate or cortisol levels, combined with factors like high patient volumes or insufficient staffing, can signal that a staff member is experiencing stress. The system looks for correlations between the biometric data and stressors, such as the timing of shifts, workload distribution, patient demand and resource availability. This early discovery is crucial because it allows healthcare management to take proactive steps to avoid more serious effects, such as burnout or tiredness.

The framework operates through seven distinct stages. Stage 1 begins with healthcare staff wearing biometric devices that continuously track stress indicators such as heart rate and HRV. Stage 2 involves real-time data collection, ensuring constant monitoring of stress levels. In Stage 3, data are transmitted to an AI-enabled centralized system for analysis and stress detection. Stage 4 assesses whether the stress threshold has been exceeded; if not, monitoring continues. Stage 5 activates the agile command hub when thresholds are surpassed, displaying stress data and resource status on a real-time dashboard. Stage 6 implements targeted interventions such as adjusting workloads, modifying schedules, or introducing wellness activities. Finally, Stage 7 closes the loop by evaluating the effectiveness of interventions and refining thresholds for continuous improvement. The system offers changes to workload distribution, shift scheduling and job allocation to produce a more sustainable work environment. For example, it may advocate changing shift times to ensure that employees are not overwhelmed or that job allocation is more equitable. Wellness initiatives, like mindfulness exercises, relaxation methods, or regular breaks, can also be incorporated into the system to help employees recover and build resilience. These long-term techniques create a work climate promoting employee well-being, lowering the likelihood of chronic stress and burnout. Finally, the framework aims to build a sustainable and resilient healthcare workforce. The solution solves acute stresses and promotes long-term well-being for healthcare workers by combining real-time monitoring, AI-driven analysis and Agile decision-making. Beyond operational benefits, the framework also strengthens organizational leadership and decision-making capabilities.

The Biometric Stress Monitoring framework significantly enhances healthcare leadership abilities by giving leaders the tools and data to proactively manage employee stress and well-being. Decision-making is a critical leadership ability that this framework helps to improve. Real-time biometric data and operational indicators enable executives and managers to make educated decisions regarding task distribution, resource allocation and stress management, ensuring prompt and successful interventions, improving problem-solving abilities because leaders may recognize stressors, such as understaffing or high patient loads and focus on alleviating them before they lead to burnout. This framework can also improve emotional intelligence and empathy in leadership. Leaders may respond with compassion and support by recognizing their employees’ physiological stress levels and the variables contributing to their stress, such as offering breaks, modifying schedules and providing more resources. This emotional awareness builds trust and promotes a supportive work atmosphere where employees feel appreciated and understood.

Technology improves leadership agility by allowing leaders to adapt rapidly to changing events in real time. Healthcare environments are dynamic and leaders must be able to respond quickly to changing patient loads, staffing shortages and other operational difficulties. Making educated judgments allows executives to maintain efficiency while boosting employee well-being, encouraging cooperation and teamwork. The data insights enable managers to collaborate directly with their staff to address stress-related concerns. Leaders participate in collaborative decision-making, giving employees a voice in resolving their issues and instilling a feeling of shared responsibility. This method also helps managers develop resilience by encouraging them to take a long-term approach to employee well-being. Leaders ensure that stress management methods are long-term and successful by constantly improving tactics based on feedback, resulting in a resilient workforce capable of addressing future issues.

Furthermore, the framework promotes responsibility and openness in leadership. Leaders are accountable for reviewing the efficacy of their initiatives and making necessary modifications, which supports a culture of accountability and continual improvement. The framework promotes strategic vision among leaders. Leaders may integrate staff well-being with performance by incorporating stress management into larger corporate goals, guaranteeing a long-term, sustainable approach to employee happiness and organizational efficiency. In this approach, the framework assists leaders in developing various key abilities, resulting in a healthier, more resilient and high-performing healthcare environment.

## Implications of the biometric stress framework for practice

The proposed framework offers transformative implications for healthcare management, addressing the critical disconnect between strategic leadership discourse and real-time workforce decision-making. Integrating biometric indicators into Agile decision-making cycles, the framework enables healthcare leaders to transition from retrospective management to proactive, anticipatory interventions. This shift facilitates task reallocation and workflow adjustments informed by real-time physiological stress signals, moving beyond reliance on delayed incident reports. Such an approach directly supports the objective of adaptability through measurable, physiologically grounded leadership actions. This framework establishes a model of continuous monitoring and micro-adjustments that empowers decision-making at the point of care. Embedding human factors engineering principles accounts for cognitive load, shift intensity and task ergonomics, transforming resilience from an abstract behavioral expectation into a systematically designed function. This alignment with Agile values structurally enables healthcare teams to enact adaptive practices effectively, bridging theory and practice through practical applications in daily operations.

The framework uses adaptive leadership by providing leaders with actionable insights into workforce stress levels. This enhanced visibility allows for regulating distress, maintaining productive tension and preventing cognitive overload, thereby mitigating performance degradation. These capabilities translate into measurable outcomes: improved staff retention, reduced burnout, enhanced decision-making precision, and more equitable workload distribution, leading to societal benefits that include improved quality of life via enhanced patient care, reduced medical errors and better workforce well-being, as healthier working conditions are associated with patient safety and overall public health improvements. Collectively, these benefits sustain high-quality patient care amidst fluctuating demands. The Biometric Stress Monitoring Framework positions healthcare organizations as learning-oriented systems by redefining resilience as a data-informed leadership capability rather than an individual trait. This evolution enables institutions to absorb strain without compromising care delivery, fostering a shift toward adaptive, evidence-based management practices.

## Future research direction

To advance the Biometric Stress Monitoring Framework, future research should focus on pilot implementations and empirical validation across diverse healthcare settings to assess its effectiveness in predicting burnout, informing adaptive workforce decisions and enhancing real-time resilience. Studies should prioritize calibrating biometric thresholds to factors such as patient load, shift duration and cognitive demand to develop sensitive decision triggers for healthcare leaders. Further exploration is warranted into integrating AI-driven analytics to personalize stress alerts and recommend micro-adjustments in staffing. Longitudinal studies are essential to evaluate the sustained impact of these interventions on staff retention, psychological safety and the cultivation of an organizational learning culture, particularly in high-pressure clinical environments.

Attention should also be directed toward assessing the framework’s scalability and interoperability in resource-intensive and resource-constrained healthcare settings, with initial validation recommended in developed nations’ resource-intensive environments, followed by adaptation to developing nations’ resource-constrained settings for broader scalability. Compatibility with existing digital health infrastructures, electronic health records and regulatory frameworks, such as HIPAA and biometric data ethics standards, must be ensured. Finally, research should investigate leadership training models incorporating biometric dashboard literacy and adaptive decision-making protocols. Such models would empower leaders to transition from traditional supervisory roles to proactive coordinators of real-time resilience, fostering sustainable healthcare systems.

## Conclusion

This study proposes a Biometric Stress Monitoring Framework. This innovative layer integrates Agile management principles, human factors engineering and Adaptive Leadership to create a real-time decision-support system for enhancing workforce resilience in healthcare settings. Biometric feedback is leveraged as actionable leadership signals; the framework enables early detection of workforce stress and facilitates micro-level adaptive interventions. This approach transforms static cultural aspirations into a measurable, dynamic management function, allowing healthcare teams to dynamically redistribute workloads, mitigate distress and maintain stability. Leadership is reframed as a situational, data-informed practice responsive to physiological stress indicators and human factors’ constraints. While the framework offers significant promise, its efficacy requires empirical validation through real-world clinical implementation, alignment with digital infrastructure and targeted development of leadership competencies. Successful adoption could redefine Agile healthcare management as a continuous, feedback-driven resilience function, fostering adaptive, psychologically safe healthcare environments that sustain workforce well-being and high-quality patient care.
